# Protective Effects of *APOE* ε2 Genotype on Cognition in Older Breast Cancer Survivors: The Thinking and Living With Cancer Study

**DOI:** 10.1093/jncics/pkab013

**Published:** 2021-01-27

**Authors:** Kathleen Van Dyk, Xingtao Zhou, Brent J Small, Jaeil Ahn, Wanting Zhai, Tim Ahles, Deena Graham, Paul B Jacobsen, Heather Jim, Brenna C McDonald, Kelly Nudelman Holohan, Sunita K Patel, G William Rebeck, James C Root, Andrew J Saykin, Harvey Jay Cohen, Jeanne S Mandelblatt, Judith E Carroll

**Affiliations:** 1 UCLA Department of Psychiatry and Biobehavioral Sciences, University of California, Los Angeles, David Geffen School of Medicine, Jane and Terry Semel Institute for Neuroscience and Human Behavior, Jonsson Comprehensive Cancer Center, Los Angeles, CA, USA; 2 Department of Biostatistics, Bioinformatics and Biomathematics, Georgetown-Lombardi Comprehensive Cancer Center, Georgetown University, Washington, DC, USA; 3 School of Aging Studies, University of South Florida, and Senior Member, Health Outcome and Behavior Program and Biostatistics Resource Core, H. Lee Moffitt Cancer Center and Research Institute at the University of South Florida, Tampa, FL, USA; 4 Department of Biostatistics, Bioinformatics, and Biomathematics, Lombardi Comprehensive Cancer Center, Georgetown University, Washington, DC, USA; 5 Department of Psychiatry and Behavioral Sciences, Memorial Sloan-Kettering Cancer Center, New York, NY, USA; 6 John Theurer Cancer Center, Hackensack, NJ, USA; 7 Division of Cancer Control and Population Sciences, Healthcare Delivery Research Program, National Cancer Institute, Bethesda, MD, USA; 8 Department of Health Outcomes and Behavior, Moffitt Cancer Center and Research Institute, University of South Florida, Tampa, FL, USA; 9 Department of Radiology and Imaging Sciences, Center for Neuroimaging, Indiana University Melvin and Bren Simon Comprehensive Cancer Center, Indiana University School of Medicine, Indianapolis, IN, USA; 10 Department of Medical and Molecular Genetics, Indiana Alzheimer’s Disease Research Center, Indiana University School of Medicine, Indianapolis, IN, USA; 11 Departments of Population Sciences and Supportive Care Medicine, City of Hope Comprehensive Cancer Center, Duarte, CA, USA; 12 Department of Neurosciences, Georgetown University School of Medicine, Georgetown University, Washington, DC, USA; 13 Departments of Psychiatry and Anesthesiology, Weill Medical College of Cornell University, New York, NY, USA; 14 Department of Radiology and Imaging Sciences, Center for Neuroimaging, Indiana Alzheimer’s Disease Research Center, and the Indiana University Melvin and Bren Simon Comprehensive Cancer Center, Indiana University School of Medicine, Indianapolis, IN, USA; 15 Center for the Study of Aging and Human Development, Duke Cancer Institute, Duke University School of Medicine, Durham, NC, USA; 16 Department of Oncology, Cancer Prevention and Control Program, Georgetown-Lombardi Comprehensive Cancer Center, Georgetown University, Washington, DC, USA; 17 Cousins Center for Psychoneuroimmunology, University of California, Los Angeles, Los Angeles, CA, USA

## Abstract

**Background:**

Cancer-related cognitive decline (CRCD) has been linked to apolipoprotein E (*APOE*) gene ε4 polymorphisms. *APOE* ε4 polymorphisms are also the strongest genetic risk for late-onset Alzheimer disease (AD), whereas ε2 polymorphisms protect against AD. However, the effects of ε2 polymorphisms on CRCD have not been evaluated.

**Methods:**

We evaluated nonmetastatic breast cancer survivors (n = 427) and matched noncancer controls (n = 407) ages 60-98 years assessed presystemic therapy from August 2010 to December 2017 with annual follow-up to 24 months. Neuropsychological assessment measured attention, processing speed, executive function, and learning and memory. Linear mixed-effects models tested the effects of having an ε2 allele (vs none) on longitudinal cognitive domain *z* scores by treatment group (chemotherapy with or without hormonal therapy, hormonal therapy, and control) controlling for covariates; participants with ε2/ε4 genotype were excluded. Sensitivity analyses examined effects of other covariates and any ε4 positivity.

**Results:**

There was an interaction with genotype for attention, processing speed, and executive functioning domain scores (Beta = 0.32, 95% confidence interval = 0.00 to 0.65); the chemotherapy group with an ε2 allele had higher scores at baseline and maintained higher scores over time compared with those without an ε2 allele, and this protective effect was not seen for other groups. There was no effect of ε2 on learning and memory domain scores.

**Conclusions:**

*APOE* ε2 polymorphisms may protect against CRCD in older breast cancer survivors receiving chemotherapy. With replication, this information could be useful for survivorship care and informing future studies of possible links to AD and defining mechanisms of protection.

Cancer-related cognitive decline (CRCD) is increasingly recognized among some breast cancer survivors, and even subtle declines can adversely affect functioning and quality of life (1-4). Efforts to identify risk factors suggest the etiology is multifactorial and may include direct effects of cancer, treatment toxicity, age, and genetic vulnerability (5-11). The apolipoprotein E (*APOE*) gene is the most commonly studied gene in CRCD (11,12). The ε4 allele is seen in roughly 25% of the population, is the strongest genetic risk factor for late-onset Alzheimer disease (AD), and has been associated with risk of CRCD in most studies (11-14), particularly after chemotherapy (15). The ε2 allele, seen in about 8% of the population (16), is considered to be protective against developing AD (17-19). However, there are no data on the potential protective effects of the ε2 allele on CRCD (11).

To fill this clinical gap, we conducted an evaluation of the role of *APOE* ε2 in longitudinal cognitive function among older breast cancer survivors and a matched noncancer control group in the Thinking and Living with Cancer (TLC) study (15,20). We hypothesized that having an ε2 allele would have the greatest protective effects in women who received chemotherapy with or without hormonal

therapy compared with those taking hormonal therapy only or controls. The results are intended to guide future research to inform practice (21), extend our results, and study possible links between CRCD and AD.

## Methods

### Design, Population, and Data Collection

We conducted a secondary analysis using data from the TLC multisite prospective study (ClinicalTrials.gov identifier: NCT03451383) of older breast cancer survivors and frequency-matched noncancer controls (15). All institutional review boards approved the protocol (NCT03451383).

We included participants recruited between August 1, 2010, and December 31, 2017, and followed until January 29, 2020; the study is ongoing. Eligible breast cancer survivors were 60 years of age or older, newly diagnosed with primary nonmetastatic breast cancer, and able to complete assessments in English; women with a history of stroke, head injury, major axis I psychiatric disorders, and neurodegenerative disorders were excluded. We also excluded women with a prior history of cancer if active treatment occurred in the 5 years prior to enrollment or included systemic therapy. Controls included friend and community controls frequency matched to survivors by age, race, education, and recruitment site; controls had the same exclusion criteria as survivors. To be included in this analysis, participants were also required to have *APOE* genotype data (available for 98.3% of survivors and 96.6% of controls).

**Figure 1.**
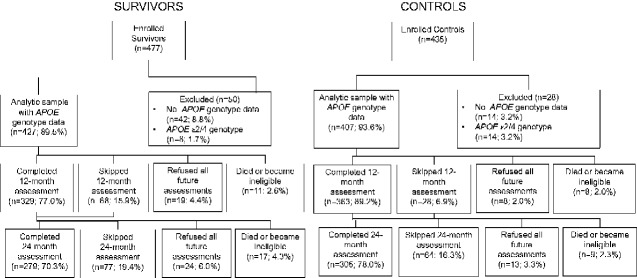
Analytic sample of older breast cancer survivors and matched noncancer controls. *APOE* = apolipoprotein E.

Participants were screened using the Mini-Mental State Examination (22) and the Wide Range Achievement Test, 4th edition (WRAT-4) Word Reading (23) subtest. Those with scores of less than 24 or below third grade equivalent reading level, respectively, were ineligible (1 survivor, 1 control). Controls who scored more than 3 standard deviations below the control mean baseline neuropsychological scores for their age and education group were ineligible post hoc (n = 8) per protocol (15). Patients were ineligible for follow-up if they developed any of the initial exclusions, and prior data were excluded if the change in eligibility occurred within 6 months of the prior follow-up visit. The final analytic sample included 427 survivors and 407 controls (see Figure 1).

Assessments were conducted by trained staff at enrollment (postsurgery, presystemic therapy for survivors) and annually through 24 months and included a structured survey, neuropsychological testing, and provision of lab specimens.

### Measures

Our primary outcomes were longitudinal scores on neurocognitive testing of 2 domains relevant to CRCD (24) and supported by previous factor analysis (15): attention, processing speed, and executive functioning (6 tests); and verbal learning and memory (5 tests) (Supplementary Table 1, available online) (25-28). Scores were transformed into *z* scores based on age and education group-matched noncancer control baseline means and standard deviations. In sensitivity analyses, we included self-reported cognition as measured on the Functional Assessment of Cancer Therapy-Cognition (29,30).

*APOE* genotype for rs7412 and rs429358 was determined using a combination of TaqMan assays (Life Technologies, Carlsbad, CA) and Fluidigm genotyping using a custom-designed 96–single-nucleotide polymorphism fingerprinting chip (Fluidigm, San Francisco, CA).

We examined several potential covariates of the relationship between cognition and genotype, including sociodemographics (age, race, marital status), psychological factors (depression, anxiety), sleep (disturbed sleep yes/no based on a 2-item measure) (31), smoking history (ever vs never), and cognitive and physical reserve. Clinical depression was defined as 16 or higher on the Center for Epidemiologic Studies Depression Scale (32), and the State-Trait Anxiety Inventory State total score measured anxiety (33). We used the WRAT-4 to measure cognitive reserve (23). A 42-item deficit accumulation index (15,34,35) was used to capture comorbidities, polypharmacy, functional ability, psychosocial factors (eg, marital status, social support, anxiety, depression, fatigue), and self-reported family history of dementia (first-degree relative) but excluded cognition. We also explored baseline cardiovascular disease (any angina, arrhythmia, myocardial infarction, and other cardiovascular diseases) as a potential confounder of the effects of *APOE* on cognition.

### Statistical Analysis

Univariable statistics summarized the relationship between baseline characteristics and *APOE* ε2 genotype (any ε2 allele vs not) and survivors and controls. The non-Finnish European population frequency for *APOE* alleles (16) was used to compare genotype distributions in our sample with those expected in the general population and assessed for statistically significant differences using Hardy-Weinberg equilibrium testing (36,37).

For multivariable analyses, we excluded participants with the ε2/ε4 genotype because any protective effect of ε2 might be attenuated by the ε4 allele (13). We used separate linear mixed models to test the effect of *APOE* ε2 genotype (any vs no ε2 allele) and treatment group (chemotherapy with or without hormonal therapy, hormonal therapy alone, or noncancer control) on longitudinal standardized *z* scores for the attention, processing speed, executive functioning, and learning and memory cognitive domains. We examined 2- and 3-way interactions of genotype, treatment group, and time. We also evaluated deficit accumulation index (which includes family history of dementia) or anxiety, depression, smoking history, time since surgery, and sleep disturbance as potential covariates. Variables were retained in the final models if they had *P* values less than .20 and face validity and improved the model goodness of fit based on Bayesian Information Criterion. All models included baseline age, race (White vs non-White), WRAT-4 score, recruitment site, and baseline deficit accumulation scores to capture variability related to factors that might affect genotype-cognition relationships. We also tested whether there were statistically significant interactions between baseline deficit accumulation scores, treatment group, and genotype (18). We considered *P* values less than .05 to be statistically significant, and all tests were 2-sided.

We also conducted several sensitivity analyses. First, we examined models that excluded participants with ε3/ε4 and ε4/ε4 genotypes to confirm that the presence of any ε4 allele did not confound results (13). Next, because cognitive declines can be subtle (38), we modeled the effects of ε2 on individual neuropsychological test scores and self-reported cognition. The *APOE* ε2 genotype has been associated with type III hyperlipoproteinemia, which may increase risk for heart disease and adversely affect cognition (39), so we tested the effects of cardiovascular-related comorbidities in lieu of baseline deficit accumulation. Finally, we evaluated model results with inverse probability weighting to address the effects of participants who dropped out or died during the course of the study. There was no statistically significant association between genotype, baseline cognition, or baseline deficits accumulation index score and subsequent dropout or death, and model results were not sensitive to missing data based on inverse probability weighting, so we report unweighted results. Analyses were conducted between October 19, 2019, and August 21, 2020, using SAS 9.4.b statistical software (SAS Institute, Cary, NC).

## Results

### Study Sample

The analytic sample remaining alive and eligible for follow-up after baseline comprised 77.0% and 70.3% survivors and 89.2% and 78.0% controls, who completed 12- and 24-month assessments, respectively. There were no statistically significant differences in baseline variables related to cognition by number of assessments completed. The majority of the sample (94%) provided specimens for genotyping and did not differ in terms of age, race, WRAT, education, or baseline cognition scores from those who did not; the control group had a smaller proportion of women with no specimens than that of either of the other 2 treatment groups (chemotherapy vs control *P* = .04; hormonal only vs control *P* = .06). The survivors and noncancer controls were demographically comparable (Table 1). Among survivors, women who received chemotherapy (with or without hormonal treatment) had more advanced stage (*P* < .001), were younger (*P* < .001), and had fewer cardiovascular comorbidities (*P* = .02) than women treated with hormonal therapy alone. Survivors selected for chemotherapy also had the highest levels of baseline anxiety and depression across the group; survivors also had higher levels of baseline sleep disturbance than controls. Participants had an overall frequency of any ε2 allele of 15.2%, similar to the expected frequency (13.5%) in White non-Hispanic populations, with no statistically significant differences among treatment groups in ε2 status (Table 2).

**Table 1.** Demographics and clinical information in the analytic sample with *APOE* genotypes excluding ε2/ε4

Variable	All survivors^a^	ε2- survivors	ε2- survivors^b^	All controls^c^	ε2- controls^b^	ε2+ controls	*P* case vs.control	*P* overalldifference acrosscase control by ε2+/-
Total No.	427	367	60	407	359	48		
Mean age (SD), y	67.9 (5.8)	68.0 (5.8)	67.4 (6.0)	67.7 (6.8)	67.8 (6.9)	67.2 (6.5)	.66	.99
Race, % (No.)							.14	.34
White	78.4 (334)	80.3 (294)	66.7 (40)	82.5 (335)	83.0 (297)	79.2 (38)		
Nonwhite	21.6 (92)	19.7 (72)*	33.3 (20)*	17.5 (71)	17.0 (61)	20.8 (10)		
Marital status, % (No.)							<.001	.76
Married	62.9 (259)	63.0 (223)	62.1 (36)	50.1 (200)	49.9 (175)	52.1 (25)		
Widowed, divorced, single	37.1 (153)	37.0 (131)	37.9 (22)	49.9 (199)	50.1 (176)	47.9 (23)		
Mean education (SD), y	15.3 (2.2)	15.3 (2.1)	15.2 (2.3)	15.5 (2.3)	15.4 (2.3)	15.8 (2.1)	.18	.45
Mean WRAT-4 score (SD)	110.4 (15.4)	110.4 (15.5)	110.5 (15.2)	111.6 (15.9)	111.3 (15.8)	113.6 (16.7)	.28	.50
Mean attention, processing speed, and executive functioning *z* score^d^ (SD)	−0.13 (0.67)	−0.12 (0.67)	−0.18 (0.66)	−0.02 (0.66)	0.00 (0.66)	−0.15 (0.63)	.02	.55
Mean learning and memory *z* score^d^ (SD)	−0.05 (0.85)	−0.05 (0.85)	−0.08 (0.82)	0.01 (0.80)	−0.00 (0.80)	0.10 (0.81)	.25	.45
Mean FACT-Cog perceived cognitive impairments^e^ (SD)	61.7 (10.6)	61.6 (10.4)	61.9 (12.1)	62.3 (8.7)	62.3 (8.7)	62.2 (8.7)	.36	.86
Chemotherapy regimen, % (No.)							—	—
Anthracycline-cyclophosphamide without taxane	6.9 (7)	7.2 (6)	5.6 (1)	0.0 (0)	—	—	—	—
Anthracycline-cyclophosphamide and taxane	46.5 (47)	49.4 (41)	33.3 (6)	0.0 (0)	—	—		
Cyclophosphamide, methotrexate, fluorouracil	11.9 (12)	8.4 (7)	27.8 (5)	0.0 (0)	—	—		
Taxane only	34.7 (35)	34.9 (29)	33.3 (6)	0.0 (0)	—	—		
AJCC v. 6 stage, % (No.)							—	—
0	12.0 (50)	13.1 (47)	5.3 (3)	0.0 (0)	—	—		
I	56.3 (234)	56.3 (202)	56.1 (32)	0.0 (0)	—	—		
II	26.4 (110)	25.1 (90)	35.1 (20)	0.0 (0)	—	—		
III	5.3 (22)	5.6 (20)	3.5 (2)	0.0 (0)	—	—		
Surgery type, % (No.)							—	—
BCS with/without RT	62.0 (263)	62.5 (228)	59.3 (35)	0.0 (0)	—	—		
Mastectomy	38.0 (161)	37.5 (137)	40.7 (24)	0.0 (0)	—	—		
Mean time since surgery to baseline (SD), d	44.3 (52.1)	43.6 (51.4)	48.5 (56.4)	—	—	—	—	—
ER status, % (No.)							—	—
Positive	88.3 (377)	87.7 (322)	91.7 (55)	0.0 (0)	—	—		
Negative	11.7 (50)	12.3 (45)	8.3 (5)	0.0 (0)	—	—		
HER2 status, % (No.)							—	—
Positive	9.8 (38)	10.6 (35)	5.3 (3)	0.0 (0)	—	—		
Negative	90.2 (349)	89.4 (295)	94.7 (54)	0.0 (0)	—	—		
Family history of dementia, % (No.)^f^							.22	.43
Yes	30.9 (132)	31.1 (114)	30.0 (18)	34.9 (142)	35.9 (129)	27.1 (13)		
No	69.1 (295)	68.9 (253)	70.0 (42)	65.1 (265)	64.1 (230)	72.9 (35)		
Smoking status, % (No.)							.91	.30
Current/former smoker	41.8 (170)	42.7 (149)	36.2 (21)	42.2 (167)	41.7 (145)	45.8 (22)		
Never smoked	58.2 (237)	57.3 (200)	63.8 (37)	57.8 (229)	58.3 (203)	54.2 (26)		
Mean comorbidities (SD)	2.6 (1.9)	2.6 (1.9)	2.7 (2.0)	2.3 (1.8)	2.4 (1.8)	2.0 (1.9)	.02	.27
Mean cardiovascular comorbidities including hypertension (SD)	0.6 (0.7)	0.7 (0.7)	0.5 (0.6)	0.5 (0.7)	0.6 (0.7)	0.5 (0.6)	.07	.70
Mean Deficits Accumulation Index^g^ (SD)	0.15 (0.08)	0.15 (0.08)	0.15 (0.09)	0.13 (0.07)	0.13 (0.07)	0.12 (0.07)	.001	.47
Depression, ≥ 16 on CES-D, % (No.)^h^	12.1 (47)	12.6 (42)	9.4 (5)	5.1 (20)	4.9 (17)	6.3 (3)	<.001	.48
Mean anxiety score (SD)^i^	28.9 (7.8)	29.0 (7.8)	28.3 (8.0)	26.5 (5.4)	26.6 (5.4)	26.4 (5.6)	<.001	.72
Sleep disturbances, yes, % (No.)	35.1 (139)	36.3 (123)	28.1 (16)	24.7 (97)	25.5 (88)	18.8 (9)	.001	.97
Attrition %, drop-out or death, % (No.)	16.6 (71)	16.1 (59)	20.0 (12)	9.3 (38)	10.0 (36)	4.2 (2)	.002	.14

a Some numbers may not add to 100% because of missing data for item; 15 survivors were missing systemic therapy data. “—” = not applicable. Non-White includes Black, Hispanic, and Asian Americans and Pacific Islanders; 1 survivor and 1 control are missing race data. *P* values based on χ^2^ or *t* tests. ε2 positive = *APOE* ε2/ε2 or ε2/ε3; ε2 negative = *APOE* ε3/ε3, ε3/ε4, ε4/ε4; AJCC = American Joint Committee on Cancer; *APOE* = apolipoprotein E; BCS = breast-conserving surgery; ER = estrogen receptor; FACT-Cog = Functional Assessment of Cancer Therapy-Cognition; RT = radiotherapy; WRAT-4 = Wide Range Achievement Test, 4th edition, Word Reading Test Standard Score.

b Statistical significance between ε2- and ε2+ participants in cases and in controls has been highlighted by single asterisks (*) if *P* ≤ .05.

c Excluding 8 cases and 14 controls who are ε2/ε4.

d Neuropsychological test scores by domain. Cognitive scores were standardized using the sample mean and standard deviation of age and education group–matched baseline controls. Hence, a score of 0 indicates a score at the mean of the control group; scores less than 0 indicate lower scores than the mean of the control group, and positive scores indicate scores higher than the control mean.

e Based on the FACT-Cog Perceived Cognitive Impairments subscale. Scores range from 0 to 72, with higher scores indicating better quality of life.

f All refusal, unknown, or missing answers to family history have been treated as “no.”

g Based on scores for baseline deficits accumulation scores. Excludes cognitive function. Scores could not be calculated if more than 10% of items were missing. Marital status, body mass index, anxiety, depression, fatigue, comorbidities, including diabetes and so on, were included in the deficit accumulation scores.

h Based on the Center for Epidemiologic Studies Depression Scale (CES-D). Depression defined by score above the cut point of 16 on the CES-D.

i Based on the State-Trait Anxiety Inventory. Scores range from 20 to 80, with higher scores reflecting more anxiety.

**Table 2.** Genotype distribution^a^

*APOE* genotype	Overall sample with known *APOE* results, % (No.) (n = 856)	Noncancer controls,% (No.) (n = 421)	Survivors receiving chemotherapy with or without hormonal treatment, % (No.) (n = 119)	Survivors receiving hormonal treatment alone, % (No.) (n = 301)
ε2/2	0.5 (4)	0.5 (2)	0.0 (0)	0.3 (1)
ε2/3	12.1 (104)	10.9 (46)	17.6 (21)	11.6 (35)
ε2/4	2.6 (22)	3.3 (14)	0.8 (1)	2.3 (7)
ε3/3	64.3 (550)	63.9 (269)	64.7 (77)	65.1 (196)
ε3/4	18.3 (157)	19.5 (82)	14.3 (17)	18.3 (55)
ε4/4	2.2 (19)	1.9 (8)	2.5 (3)	2.3 (7)

a *APOE* = apolipoprotein E

### Effect of *APOE* ε2 Allele on Cognitive Outcomes

**Figure 2.**
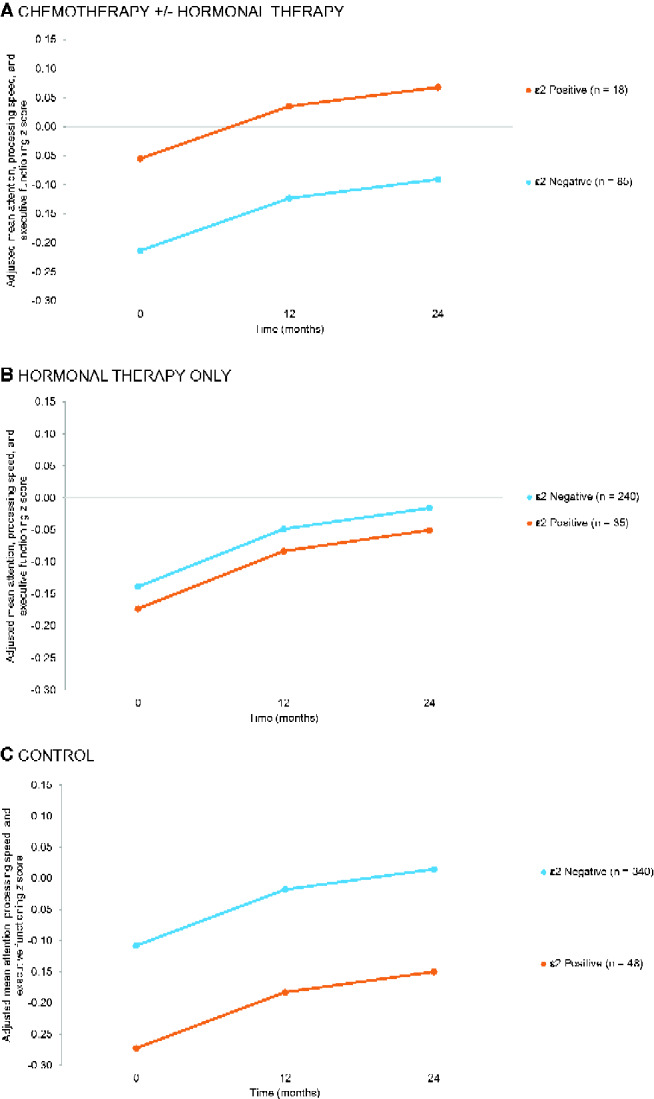
Impact of *APOE* ε2 genotype on adjusted longitudinal scores on attention, processing speed, and executive functioning *z* scores among older breast cancer survivors (n = 378) and noncancer controls (n = 388) excluding ε2/4 genotype 1. Results for **(A)** chemotherapy with and without hormonal therapy, **(B)** hormonal therapy only, and **(C)** controls are shown. Supplementary Tables 11 and 12 (available online) provide adjusted mean attention, processing speed, and executive functioning *z* scores over time and post hoc group comparisons.

We found an interaction between genotype and chemotherapy (vs control) on adjusted cognition scores for the attention, processing speed, and executive function domain (*P* = .05); however, an overall interaction (*df* = 2) between genotype and treatment group was not statistically significant (*P* = .13). In the chemotherapy group, those with an ε2 allele had higher attention, processing speed, and executive function domain scores across all time points compared with those without an ε2 allele, and this effect was not seen in the other groups (Table 3 and Figure 2). Post hoc comparisons showed non-statistically significant 0.16 of a standard deviation difference in baseline attention, processing speed, and executive functioning scores between ε2 carriers and noncarriers in the chemotherapy group (*P* = .26; see Supplementary Table 2, available online).

**Table 3.** Impact of *APOE* ε2 genotype on adjusted longitudinal scores on objective cognition test domains and FACT-Cog Perceived Cognitive Impairment Scores among older breast cancer survivors (n = 412) and noncancer controls (n = 407) excluding ε2/4 genotype^a^^,b^

Term	Final models with baseline deficits accumulation		
	Attention, processing speed, and executive function *z* score	Learning and memory *z* score	FACT-Cog 18-item perceived cognitive impairment score
*APOE* genotype			
*P*	.83	.64	.91
Any ε2 vs no ε2 allele, Beta (95% CI)	-0.16 (-0.33 to -0.00)*	0.05 (-0.16 to 0.25)	0.63 (-1.87 to 3.14)
Group			
*P*	.75	.69	.20
Chemotherapy with or without HT vs control, Beta (95% CI)	-0.11 (-0.24 to 0.02)	-0.05 (-0.22 to 0.11)	-1.21 (-3.22 to 0.81)
Hormonal vs control, Beta (95% CI)	-0.03 (-0.12 to 0.06)	0.01 (-0.11 to 0.12)	-1.01 (-2.43 to 0.40)
Time			
*P*	<.001	<.001	.07
12 months vs baseline, Beta (95% CI)	0.09 (0.06 to 0.12)**	0.20 (0.15 to 0.24)**	-0.60 (-1.26 to 0.06)
24 months vs baseline, Beta (95% CI)	0.12 (0.09 to 0.16)**	0.19 (0.14 to 0.24)**	-0.76 (-1.47 to -0.05)*
Interaction of group and genotype			
*P*	.13	.95	.78
Any ε2 allele and chemotherapy vs no ε2 allele, control, Beta (95% CI)	0.32 (0.00 to 0.65)*	-0.05 (-0.46 to 0.36)	-1.78 (-6.80 to 3.24)
Any ε2 allele and hormonal therapy vs no ε2 allele, control, Beta (95% CI)	0.13 (-0.12 to 0.38)	0.02 (-0.30 to 0.34)	-0.47 (-4.41 to 3.47)
Baseline deficits accumulation per 0.01 points, Beta (95% CI)	-0.01 (-0.02 to -0.01)**	0.00 (-0.00 to 0.01)	-0.29 (-0.37 to -0.21)**
Model Fit—BIC	2501.5	3742.6	13374.7

a Results from mixed linear models; model fit was assessed using the Bayesian Information Criteria (BIC) score; lower scores indicate better fit. This primary analysis includes women with *APOE* ε2/2, ε2/3, ε3/3, ε3/4, or ε4/4 genotypes, grouped as having any vs no ε2 allele; women with ε2/4 genotype are excluded (n = 8 survivors and 14 controls). Each covariate adjusted for the effects of the other variables, time, interactions, and age, race, WRAT score, and recruitment site. Comparable models further excluding genotypes ε3/4 and ε4/4 are included in secondary analyses in Supplementary Table 3 (available online). *APOE* = apolipoprotein E; CI = confidence interval; FACT-Cog = Functional Assessment of Cancer Therapy-Cognition.

b The inclusion of terms for comorbidities or cardiovascular disease or hyperlipidemia (instead of deficits accumulation scores) did not improve model fit so were not used. Because depression and anxiety only modestly altered model fit, they were not statistically significant factors and did not meaningfully alter results; they were not retained in the final models. Smoking and sleep disturbance were not related to group, ε2 or cognition, and were not included in the final model. Family history of dementia was included in the deficits accumulation index. Two- and 3-way interactions of ε2 or treatment group, deficits accumulation, and time were not statistically significant and were not retained in the models. Participants with missing baseline covariates or outcomes would be excluded from the model.

**Figure 3.**
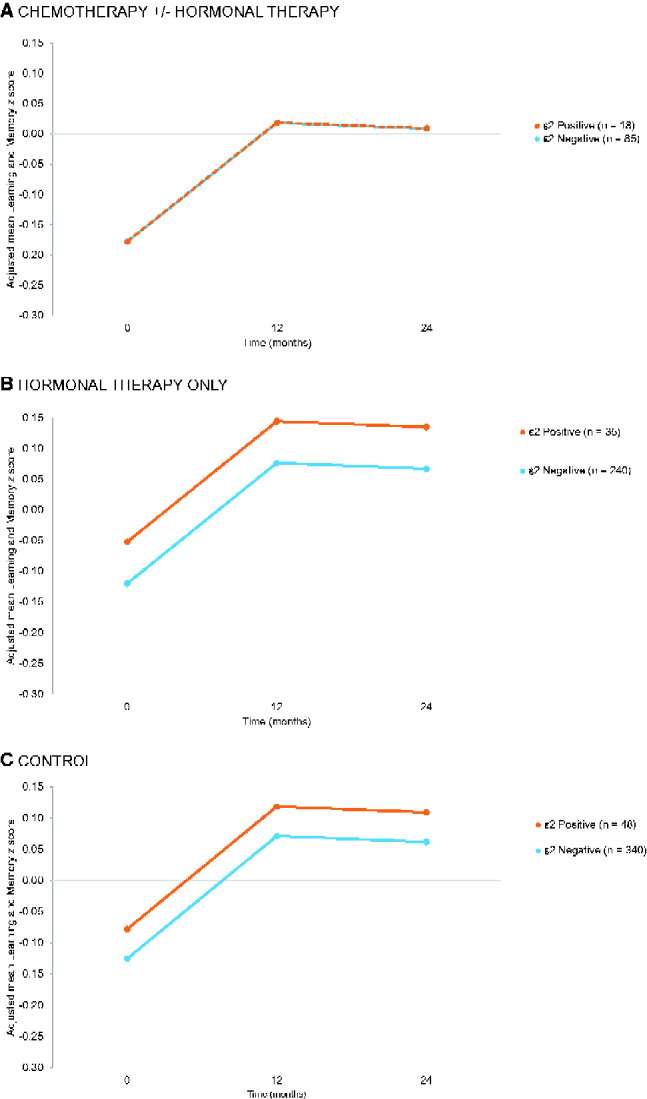
Impact of *APOE* ε2 genotype on adjusted longitudinal scores on learning and memory *z* scores among older breast cancer survivors (n = 378) and noncancer controls (n = 388) excluding ε2/4 genotype. Results for **(A)** chemotherapy with and without hormonal therapy, **(B)** hormonal therapy only, and **(C)** controls are shown. Supplementary Tables 12 and 13 (available online) provide adjusted mean learning and memory *z* scores over time and post hoc group comparisons.

Contrary to expectation, controls with an ε2 allele had lower attention, processing speed, and executive functioning scores than those without an ε2 allele (*P* = .047) across timepoints. There was no effect of ε2 genotype on learning and memory (Figure 3). Results were unchanged if anxiety, depression, or sleep disturbance was considered (Supplementary Tables 3-5, available online). Smoking history, family history of dementia, and time since surgery were not statistically significant contributors to the models, did not change the genotype-cognition results, and were not included in final models.

### Sensitivity Analysis

When we excluded participants with ε3/ε4 and ε4/ε4 genotypes, similar results were obtained (Supplementary Tables 6-8, available online). We also examined models of each constituent neuropsychological test comprising the attention, processing speed, and executive functioning domain. The effect of the ε2 genotype on cognition by treatment group tended to be driven by 2 of the 6 tests (COWAT, *P* = .02; Trail Making B, *P* = .09; Supplementary Table 9, available online). Additionally, we found no relationships between ε2 and self-reported cognition (Table 3;Supplementary Table 8, available online). If we considered the effect of mean baseline cardiovascular comorbidities instead of deficit accumulation score, we saw a similar pattern to the primary analyses, and the model fit indices were not improved compared with initial models (Supplementary Table 10, available online).

## Discussion

To our knowledge, this is the first study to examine the effect of the *APOE* ε2 allele on cognitive outcomes of cancer survivors. We found that older breast cancer survivors who were ε2 allele carriers who received chemotherapy had better cognitive performance on tests of attention, processing speed, and executive functioning before systemic therapy, and this stronger performance was maintained over 24 months. The observed protective effect was not seen among survivors on hormonal therapy or noncancer controls. Although the observed effect was of small magnitude, it may nonetheless be clinically meaningful given the subtle neurocognitive changes associated with CRCD. Genotype was not related to tests of learning and memory in any group. Results were very similar when we excluded all participants with an *APOE* ε4 allele. Neither mood, history of smoking, family history of dementia, nor time since surgery affected results. The lack of an ε2 protective effect in the noncancer control group was not explained by deficit accumulation, number of comorbidities, cardiovascular comorbidities, or other variables.

Most prior genetic studies of CRCD in breast cancer survivors have focused on *APOE* ε4. We expected that, compared with noncarriers, survivors and noncancer controls with an ε2 allele would have better cognition over time, with more pronounced protective effects for survivors because of cancer-related toxicities. However, we found that having an ε2 allele was only protective for survivors selected for chemotherapy, and the source of this effect was higher cognitive function prior to systemic therapy that then persisted over time after treatment exposure. These effects were not explained by younger age or lower comorbidities or deficits in survivors selected for chemotherapy compared with hormonal therapy alone. These findings may suggest that having an ε2 allele promoted cognitive reserve and buffered against cognitive decline in the face of challenges of high tumor burden and/or exposure to chemotherapy-related toxicities (9,40).

The *APOE* gene has pleotropic effects, although the exact mechanisms by which *APOE* genotypes affect CRCD (and AD) are largely undetermined. However, *APOE* ε2 promotes anti-inflammatory and anti-oxidant processes, supports synaptic plasticity, and protects against aging-related cognitive decline, whereas ε4 confers risk for cognitive decline (18,41-44). Thus, it is reasonable that survivors who were ε2 carriers and exposed to chemotherapy were the most protected from cognitive loss because cancer and chemotherapy are associated with DNA damage and inflammation. An alternative explanation for our result could be that oncologists are selecting older patients for treatment based on their clinical assessment of ability to survive long enough to benefit from chemotherapy (45,46), and our results may reflect unmeasured factors related to this selection bias.

Similar to prior reports from our group and others (5,9,12,15), we found that *APOE* genotype was statistically significantly associated with differences in the domain of attention, processing speed, and executive functioning but not learning and memory. However, the impact of *APOE* genotype on cognitive performance is small, therefore, it is possible that despite our relatively large sample size, we were unable to detect small effects of the ε2 genotype on learning and memory. Indeed, only 2 of the 6 tests of attention, processing speed, and executive function were related to the genotype-cognition interaction observed in the aggregate domain score. These 2 tests are arguably more demanding on executive processes than the others and thus may provide better sensitivity to subtle effects. There is a growing consensus that refining cognitive measurement sensitivity will increase the likelihood of signal detection for clinically meaningful effects in cancer populations (47).

Interestingly, we did not observe any genotype effects on self-reported cognitive function. Because the benefit of ε2 was observed at study entry and prior to chemotherapy treatment, differences in cognitive function may be more long-standing than those typically captured by the Functional Assessment of Cancer Therapy-Cognition (ie, acute, noticeable changes related to cancer treatment). Prior work in this cohort has similarly detected effects of the ε4 genotype only on neuropsychological testing and not on self-report (15). CRCD is likely multifactorial and measured using both objective and subjective means, and the association of self-reported cognition to genetic factors requires further study.

A strength of our study is having a control group, allowing comparison of longitudinal findings among breast cancer survivors to those in a normative sample without cancer. We were surprised by the finding of a relative disadvantage of ε2 positivity in our noncancer control group across timepoints. It is unclear how to interpret this finding, because survivors and noncancer controls were well-matched at enrollment, and accounting for covariates that differed between the treatment groups such as anxiety and depression did not affect results. Because the *APOE* ε2 genotype has been associated with type III hyperlipoproteinemia (39) and is linked to cardiovascular health (48,49), we also evaluated cardiovascular comorbidities, and these did not markedly change the results. It is possible that the exclusion of participants with neurodegenerative disease had a differential effect on results for survivors and controls. It will be important to understand the broader effects of ε2 on health and cognition in cancer populations and integrate evidence from noncancer studies. Furthermore, these unexpected findings in our control group could signal the need to attend to specific genotype in study design or analysis of comparison samples. Overall, the effects of ε2 may dynamically influence risks and benefits across multiple outcomes, which are yet to be fully appreciated.

Other clinically relevant findings include the fact that similar to past studies of CRCD (15) and current models of dementia risk (50), we found that *APOE* genotypes do not correspond to a family history of dementia. Further, baseline mood symptoms and smoking history failed to explain the relationship between ε2 status and neuropsychological outcomes, suggesting these are distinct clinical outcomes, consistent with current multifactorial theories of CRCD (3,6,31).

There are several limitations to this study that should be considered in evaluating our results. First, this was a secondary unplanned analysis, and although a protective effect of ε2 on cognitive function in cancer survivors is biologically plausible and consistent with the AD literature (17-19), it will be important to replicate our findings in diverse settings and populations (51,52). Second, our power to detect small effects was limited because the ε2 allele is infrequent (13). Very few women who received chemotherapy had the ε2/ε2 or ε2/ε3 genotype, and an even lower percentage of women receiving only hormonal therapy had either genotype, underscoring the need for further study across treatment exposures. We were also not able to examine dose-response effects of the number of ε2 alleles or the effects among different chemotherapy regimens. Third, follow-up of more than 24 months may be needed to evaluate the role of genotype on later risk of cognitive decline. Finally, ε2 may protect aspects of cognition not captured in our neuropsychological battery.

Our result that the *APOE* ε2 allele may confer protection against cognitive decline for cancer survivors selected to receive chemotherapy adds a new dimension to the body of evidence supporting a role of *APOE* genotype broadly and strengthens evidence suggesting parallels between CRCD and AD (9,12,15,31,53). This idea is supported by indirect evidence, including neuroimaging studies showing that breast cancer survivors and individuals with AD have abnormalities in similar brain regions (54,55) and overlap in the cognitive domains affected (9,56). There is also increasing evidence showing that inflammatory pathways are likely involved in the development of both conditions and anti-inflammatory activity varies by *APOE* genotype (18,57-62). Because our results were unchanged when we excluded all ε4 carriers, our ε2 findings are not merely the inverse of the ε4 findings previously reported (15) and are consistent with the unique effects of each variant described in the AD literature (18). Overall, this study is the first to demonstrate a potential protective effect of the ε2 allele on CRCD in breast cancer survivors, and the need for replication is emphasized. Determining genetic protection from or risk for CRCD remains a priority to help patients understand their risk for these symptoms and improve prevention, assessment, and informed treatment decisions (5,21).

## Funding

This research was supported by the National Cancer Institute at the National Institutes of Health grants K08CA241337 to KVD and R01CA129769 and R35CA197289 to JSM. This study was also supported in part by National Institute of Aging at the National Institutes of Health grant R01AG068193 to JSM and AJS. This research was also supported in part by the National Cancer Institute at the National Institutes of Health grant P30CA51008 to Georgetown-Lombardi Comprehensive Cancer Center for support of the Biostatistics and Bioinformatics Resource and the Non-Therapeutic Shared Resource. The work of AJS and BCM was supported in part by the National Cancer Institute, National Institute of Aging, and National Library of Medicine at the National Institutes of Health grants R01CA244673, P30CA082709, P30AG10133, R01AG19771, and R01LM01136. GWR was supported in part by National Institute of Aging at the National Institutes of Health grant R01AG067258. TA was supported in part by National Cancer Institute at the National Institutes of Health grants R01CA172119 and P30CA008748. The work of JEC was supported in part by the American Cancer Society Research Scholars grant 128660-RSG-15-187-01-PCSM and the National Cancer Institute at the National Institutes of Health grant R01CA237535. HJC was supported in part by the National Institute of Aging at the National Institutes of Health grant P30AG028716 for the Duke Pepper Center. SKP was supported in part by the American Cancer Society Research Scholar grant RSG-17-023-01.

## Notes

**Role of the funders:** The study sponsors did not play any role in the study design, data collection, analysis or interpretation; manuscript preparation; or submission.

**Disclosures:** DG reports COTA stock ownership. HJ reports grant funding from Kite Pharmaceuticals and consultant to RedHill BioPharma, Janssen Scientific Affairs and Merck. KNH reports salary and projects are supported by grants from the NIH (U24AG021886, U24NS095871, U01AG057195, U01AG24904), a sub-award from a collaborative clinical trial between the ADCS and Biohaven Pharmaceuticals (UCSD sub-award, IU site, 107214854), nonprofit entities (Michael J. Fox, 10457.04, and Lilly Endowment Inc, 20091568000), US Army Medical Research (W81XWH1820047), and US Department of Defense (4638). AJS reports relationships with Bayer Oncology (advisory board unrelated to this work) and Springer Nature (editorial office support unrelated to this work). All other authors report no disclosures.

**Disclaimer:** The content is solely the responsibility of the authors and does not represent the official views of the National Institutes of Health or other funding agencies.

**Acknowledgements:** We would like to thank the participants in the Thinking and Living with Cancer study for their sharing of their time and experiences; without their generosity, this study would not have been possible. We are also indebted to Sherri Stahl, Naomi Greenwood, Margery London, and Sue Winarsky, who serve as patient advocates from the Georgetown Breast Cancer Advocates, for their insights and suggestions on study design and methods to recruit and retain participants. We thank the Thinking and Living with Cancer study staff who contributed by ascertaining, enrolling, and interviewing participants. The work of Paul Jacobsen was done while he was at Moffitt Cancer Center.

**Author contributions:** Kathleen Van Dyk: Conceptualization, writing-original draft, and writing- review and editing. Xingtao Zhou: Data curation, formal analysis, and writing-original draft, writing-review and editing, visualization. Brent J. Small: Writing-original draft, data curation, formal analysis, and writing-review and editing. Jaeil Ahn: Data curation, formal analysis, writing-original draft, and writing-review and editing. Wanting Zhai: Data curation, formal analysis, writing-original draft, and writing-review and editing. Tim Ahles: Funding acquisition, investigation, methodology, project administration, resources, supervision, or validation, and writing-review and editing. Deena Graham: Funding acquisition, investigation, methodology, project administration, resources, supervision, or validation, and writing-review and editing. Paul B. Jacobsen: Funding acquisition, and writing-review and editing. Heather S. Jim: Funding acquisition, investigation, methodology, project administration, resources, supervision, or validation, and writing-review and editing. Brenna C. McDonald: Funding acquisition, investigation, methodology, project administration, resources, supervision, or validation, and writing-review and editing. Kelly N Holohan: Data curation, formal analysis, and writing-review and editing. Sunita K. Patel: Funding acquisition, investigation, methodology, project administration, resources, supervision, or validation, and writing-review and editing. G. William Rebeck: Conceptualization and writing-review and editing. James C. Root: Funding acquisition, investigation, methodology, project administration, resources, supervision, or validation, and writing-review and editing. Andrew J. Saykin: Funding acquisition, investigation, methodology, project administration, resources, supervision, or validation, and writing-review and editing. Harvey J. Cohen: Conceptualization and writing-review and editing. Jeanne S. Mandelblatt: Conceptualization, writing-original draft, funding acquisition, investigation, methodology, project administration, resources, supervision, or validation, and writing-review and editing. Judith E. Carroll: Conceptualization, writing-original draft, and writing- review and editing.

## Data Availability

The data underlying this article will be shared on reasonable request to the corresponding author using the Thinking and Living with Cancer data use/data sharing protocols available upon request.

## Supplementary Material

pkab013_Supplementary_Data
