# Supplementary material for: Protective Effects of APOE ε2 Genotype on Cognition in Older Breast Cancer Survivors: The Thinking and Living With Cancer Study
Source: JNCI Cancer Spectr. 2021 Jan 27;5(2):pkab013. doi: 10.1093/jncics/pkab013 (PMC7962698; doi:10.1093/jncics/pkab013)
Supplement: pkab013_Supplementary_Data [file pkab013_supplementary_data.pdf]

SUPPLEMENTARY MATERIALS

Supplementary Table 1: Raw Baseline Neuropsychological Testing Scores of Pretreatment Cognitive Performance Among Older Breast Cancer Survivors (N=412) and Non-Cancer Controls (N=407) Excluding ε2/4 Genotype

Domain/Test[a]	Study Group, Mean (SD)			
	Controls (N=407)	Survivors (N=427)	Survivor Chemotherapy (N=118)	Survivor Hormonal Therapy (N=294)
APE				
Digits Forward[b]	8.42 (2.38)	8.34 (2.34)	8.27 (2.15)	8.42 (2.44)
Digits Backward[b]	4.70 (2.28)	4.54 (2.13)	4.80 (2.12)	4.49 (2.16)
Trail Making A[c]	35.79 (12.28)	37.48 (14.36)	36.61 (14.13)	37.91 (14.70)
Trail Making B[c]	82.88 (40.93)	90.71 (46.08)	84.35 (47.11)	93.05 (46.14)
Controlled Oral Word Association Test[d]	43.70 (12.71)	41.18 (12.03)	41.64 (12.54)	41.21 (12.01)
Digit Symbol Test raw score[e]	54.00 (10.33)	51.96 (11.53)	52.64 (11.24)	51.79 (11.65)
LM				
Logical Memory I[f]	13.19 (3.73)	12.80 (3.71)	12.90 (3.73)	12.82 (3.66)
Logical Memory II[g]	11.91 (3.91)	11.67 (3.99)	11.97 (3.58)	11.61 (4.13)
NAB List A Immediate Recall[h]	23.37 (4.60)	22.79 (5.01)	23.07 (4.82)	22.75 (5.16)
NAB List A Short Delay[i]	7.72 (2.50)	7.48 (2.66)	7.48 (2.41)	7.56 (2.76)
NAB List A Long Delay raw score[j]	7.76 (2.65)	7.55 (2.69)	7.53 (2.71)	7.63 (2.66)

[a]With the exception of Trail Making A and B, higher raw scores reflect better performance. Abbreviations: APE, attention, processing speed, and executive function; COWAT, Controlled Oral Word Association Test; LM, learning and memory; NAB, Neuropsychological Assessment Battery.
[b]Number of sequences correctly recalled.
[c]Time in seconds.
[d]Number of words given in allowed time.
[e]Number of symbols produced in allowed time.
[f]Number of pieces of a story recalled immediately.
[g]Number of pieces of a story recalled after delay.
[h]Sum of three trials of word recall.
[i]Number of words recalled after short delay.
[j]Number of words recalled after long delay.

Supplementary Table 2 post-hoc group comparisons of attention, processing speed, and executive functioning z-scores

Post-hoc group comparisons		
ε2+ chemo vs. ε2- chemo	---	0.16 (-0.12, 0.44), p=.26
ε2+ hormonal vs. ε2- hormonal	---	-0.03 (-0.23, 0.16), p=.73
ε2+ control vs. ε2- control	---	-0.16 (-0.33, -0.00), p=.047
ε2+ chemo vs. ε2+ hormonal	---	0.12 (-0.19, 0.43), p=.46
ε2+ chemo vs. ε2- hormonal	---	0.08 (-0.18, 0.35), p=.53
ε2+ chemo vs. ε2+ control	---	0.22 (-0.08, 0.51), p=.15
ε2+ chemo vs. ε2- control	---	0.05 (-0.21, 0.31), p=.69

Supplementary Table 3. Impact of APOE ε2 Genotype on Adjusted Longitudinal Scores on Attention, Processing Speed, and Executive Functioning Domain Controlling for Baseline Deficits Accumulation Among Older Breast Cancer Survivors (N=412) and Non-Cancer Controls (N=407) Excluding ε2/4 Genotype[a]

	Attention, Processing Speed and Executive Functioning z-score			
	Base Model	Adding anxiety to final model[b]	Adding depression to final model[c]	Adding sleep disturbance to final model
	Beta (95% CI) or p-value			
APOE Genotype	p=.47	p=.72	p=.95	p=.87
Any ε2 vs. no ε2 allele	-0.15 (-0.32, 0.01)	-0.19 (-0.35, -0.02)	-0.17 (-0.33, -0.00)	-0.16 (-0.32, 0.00)
Group	p=1.00	p=.61	p=.69	p=.78
Chemotherapy +/- HT vs. control	-0.10 (-0.23, 0.02)	-0.09 (-0.22, 0.04)	-0.12 (-0.26, 0.01)	-0.11 (-0.24, 0.02)
Hormonal vs. control	-0.07 (-0.16, 0.02)	-0.03 (-0.12, 0.06)	-0.03 (-0.12, 0.06)	-0.03 (-0.13, 0.06)
Time	p<.001	p<.001	p<.001	p<.001
12 months vs. baseline	0.08 (0.05, 0.12)	0.09 (0.06, 0.12)	0.09 (0.06, 0.12)	0.09 (0.06, 0.12)
24 months vs. baseline	0.12 (0.09, 0.16)	0.12 (0.09, 0.16)	0.12 (0.09, 0.16)	0.12 (0.09, 0.16)
Interaction of group and genotype	p=.37	p=.11	p=.10	p=.14
Any ε2 allele and chemotherapy vs. no ε2 allele, control	0.19 (-0.12, 0.50)	0.33 (0.01, 0.66)	0.34 (0.01, 0.67)	0.32 (-0.00, 0.64)
Any ε2 allele and hormonal therapy vs. no ε2 allele, control	0.14 (-0.11, 0.39)	0.16 (-0.10, 0.41)	0.17 (-0.09, 0.43)	0.13 (-0.12, 0.39)
Baseline deficits accumulation per 0.01 points	---	-0.01 (-0.01, -0.00)	-0.01 (-0.02, -0.01)	-0.01 (-0.02, -0.01)
Anxiety per 1 point	---	-0.00 (-0.01, 0.00)	---	---
Depression (depressed vs. not depressed)	---	---	0.06 (-0.08, 0.20)	---
Sleep disturbance (sleep disturbed vs. not sleep disturbed)	---	---	---	0.04 (-0.04, 0.13)
Model Fit - BIC	2687.5	2482.6	2460.1	2503.7

[a]Results from mixed linear models; model fit was assessed using the Bayesian Information Criteria (BIC) score; lower scores indicate better fit. This primary analysis includes women with APOE ε2/2, ε2/3, ε3/3, ε3/4, or ε4/4 genotypes, grouped as having any vs no ε2 allele; women with ε2/4 genotype are excluded (n=8 survivors and 14 controls). Each covariate adjusted for the effects of the other variables, time, interactions, and age, race, WRAT score and recruitment site.

[b]The frailty item on anxiety was excluded from the baseline deficits accumulation in the model including anxiety.

[c]The frailty item on depression was excluded from the baseline deficits accumulation in the model including depression.

Supplementary Table 4 Impact of APOE ε2 Genotype on Adjusted Longitudinal Scores on Learning and Memory Domain Controlling for Baseline Deficits Accumulation Among Older Breast Cancer Survivors (N=412) and Non-Cancer Controls (N=407) Excluding ε2/4 Genotype[a]

	Learning and Memory z-score			
	Base Model	Adding anxiety to final model[b]	Adding depression to final model[c]	Adding sleep disturbance to final model
	Beta (95% CI) or p-value			
APOE Genotype	p=.68	p=.67	p=.39	p=.67
Any ε2 allele vs. no ε2	0.04 (-0.17, 0.24)	0.07 (-0.14, 0.28)	0.05 (-0.15, 0.26)	0.04 (-0.16, 0.25)
Group	p=.85	p=.73	p=.73	p=.70
Chemotherapy +/- HT vs. control	-0.02 (-0.18, 0.13)	-0.03 (-0.20, 0.14)	-0.04 (-0.20, 0.13)	-0.05 (-0.22, 0.11)
Hormonal vs. control	-0.02 (-0.13, 0.09)	0.01 (-0.11, 0.13)	0.02 (-0.09, 0.14)	0.01 (-0.11, 0.13)
Time	p<.001	p<.001	p<.001	p<.001
12 months vs. baseline	0.19 (0.15, 0.24)	0.20 (0.15, 0.25)	0.20 (0.15, 0.24)	0.20 (0.15, 0.24)
24 months vs. baseline	0.20 (0.15, 0.25)	0.19 (0.14, 0.24)	0.19 (0.14, 0.24)	0.19 (0.14, 0.24)
Interaction of group and genotype	p=.89	p=.91	p=.95	p=.96
Any ε2 allele and chemotherapy vs. no ε2 allele, control	-0.06 (-0.45, 0.33)	-0.09 (-0.50, 0.32)	0.00 (-0.41, 0.42)	-0.04 (-0.45, 0.37)
Any ε2 allele and hormonal therapy vs. no ε2 allele, control	0.04 (-0.28, 0.36)	-0.00 (-0.33, 0.32)	0.05 (-0.28, 0.38)	0.02 (-0.30, 0.34)
Baseline deficits accumulation per 0.01 points	---	0.00 (-0.00, 0.01)	0.00 (-0.00, 0.01)	0.00 (-0.00, 0.01)
Anxiety per 1 point	---	-0.01 (-0.01, 0.00)	---	---
Depression (depressed vs. not depressed)	---	---	-0.12 (-0.30, 0.06)	---
Sleep disturbance (sleep disturbed vs. not sleep disturbed)	---	---	---	-0.05 (-0.15, 0.06)
Model Fit - BIC	3991.9	3716.8	3689.0	3742.4

[a]Results from mixed linear models; model fit was assessed using the Bayesian Information Criteria (BIC) score; lower scores indicate better fit. This primary analysis includes women with APOE ε2/2, ε2/3, ε3/3, ε3/4, or ε4/4 genotypes, grouped as having any vs no ε2 allele; women with ε2/4 genotype are excluded (n=8 survivors and 14 controls). Each covariate adjusted for the effects of the other variables, time, interactions, and age, race, WRAT score and recruitment site.

[b]The frailty item on anxiety was excluded from the baseline deficits accumulation in the model including anxiety.

[c]The frailty item on depression was excluded from the baseline deficits accumulation in the model including depression.

Supplementary Table 5 Impact of APOE ε2 Genotype on Adjusted Longitudinal Scores on FACT-Cog Percieved Cognitive Impairment Controling for Baseline Deficits Accumulation Among Older Breast Cancer Survivors (N=412) and Non-Cancer Controls (N=407) Excluding ε2/4 Genotype[a]

	FACT-Cog 18-item Perceived Cognitive Impairment Score			
	Base Model	Adding anxiety to final model[2]	Adding depression to final model[3]	Adding sleep disturbance to final model
	Beta (95% CI) or p-value			
APOE Genotype	p=.99	p=.67	p=.98	p=.79
Any ε2 allele vs. no ε2	0.86 (-1.70, 3.43)	0.98 (-1.48, 3.44)	0.79 (-1.67, 3.25)	0.53 (-1.97, 3.02)
Group	p=.04	p=.32	p=.47	p=.26
Chemotherapy +/- HT vs. control	-1.62 (-3.61, 0.37)	-0.01 (-1.98, 1.97)	-0.30 (-2.32, 1.72)	-1.07 (-3.08, 0.93)
Hormonal vs. control	-1.70 (-3.12, -0.28)	-0.67 (-2.04, 0.71)	-0.76 (-2.16, 0.64)	-0.78 (-2.19, 0.64)
Time	p=.049	p=.05	p=.04	p=.07
12 months vs. baseline	-0.58 (-1.23, 0.08)	-0.62 (-1.28, 0.05)	-0.69 (-1.36, -0.03)	-0.60 (-1.27, 0.06)
24 months vs. baseline	-0.83 (-1.53, -0.13)	-0.82 (-1.53, -0.11)	-0.79 (-1.50, -0.08)	-0.76 (-1.47, -0.06)
Interaction of group and genotype	p=.45	p=.45	p=.70	p=.79
Any ε2 allele and chemotherapy vs. no ε2 allele, control	-2.87 (-7.82, 2.08)	-3.11 (-7.98, 1.76)	-2.15 (-7.16, 2.87)	-1.69 (-6.69, 3.30)
Any ε2 allele and hormonal therapy vs. no ε2 allele, control	0.31 (-3.70, 4.31)	-1.06 (-4.89, 2.77)	-0.32 (-4.25, 3.62)	-0.67 (-4.60, 3.25)
Baseline deficits accumulation per 0.01 points	---	-0.22 (-0.30, -0.15)	-0.22 (-0.30, -0.14)	-0.27 (-0.35, -0.19)
Anxiety per 1 point	---	-0.33 (-0.42, -0.24)	---	---
Depression (depressed vs. not depressed)	---	---	-6.33 (-8.50, -4.16)	---
Sleep disturbance (sleep disturbed vs. not sleep disturbed)	---	---	---	-2.00 (-3.32, -0.67)
Model Fit - BIC	13820.6	13185.6	13154.2	13344.7

[a]Results from mixed linear models; model fit was assessed using the Bayesian Information Criteria (BIC) score; lower scores indicate better fit. This primary analysis includes women with APOE ε2/2, ε2/3, ε3/3, ε3/4, or ε4/4 genotypes, grouped as having any vs no ε2 allele; women

with ε2/4 genotype are excluded (n=8 survivors and 14 controls). Each covariate adjusted for the effects of the other variables, time, interactions, and age, race, WRAT score and recruitment site.

[b]The frailty item on anxiety was excluded from the baseline deficits accumulation in the model including anxiety.

[c]The frailty item on depression was excluded from the baseline deficits accumulation in the model including depression.

Supplementary Table 6. Impact of APOE ε2 Genotype on Adjusted Longitudinal Scores on Attention, Processing Speed, and Executive Functioning Domain Controlling for Baseline Deficits Accumulation, Anxiety, Depression, or None Among Older Breast Cancer Survivors (N=330) and Non-Cancer Controls (N=317) Excluding All ε4 Carriers[a]

	Attention, Processing Speed and Executive Functioning z-score			
	Base Model	Final Model with baseline deficits accumulation	Adding anxiety to final model[b]	Adding depression to final model[c]
	Beta (95% CI) or p-value			
APOE Genotype	p=.51	p=.79	p=.70	p=.98
Any ε2 allele vs. no ε2	-0.14 (-0.31, 0.03)	-0.16 (-0.33, 0.01)	-0.18 (-0.35, -0.01)	-0.16 (-0.33, 0.01)
Group	p=.98	p=.68	p=.55	p=.59
Chemotherapy +/- HT vs. control	-0.10 (-0.24, 0.04)	-0.10 (-0.25, 0.05)	-0.09 (-0.24, 0.06)	-0.11 (-0.27, 0.04)
Hormonal vs. control	-0.06 (-0.16, 0.05)	-0.01 (-0.11, 0.10)	-0.00 (-0.11, 0.10)	-0.01 (-0.11, 0.10)
Time	p<.001	p<.001	p<.001	p<.001
12 months vs. baseline	0.10 (0.06, 0.13)	0.11 (0.07, 0.14)	0.11 (0.07, 0.14)	0.11 (0.07, 0.14)
24 months vs. baseline	0.12 (0.08, 0.16)	0.12 (0.08, 0.16)	0.12 (0.08, 0.16)	0.12 (0.08, 0.16)
Interaction of group and genotype	p=.44	p=.18	p=.15	p=.14
Any ε2 allele and chemotherapy vs. no ε2 allele, control	0.18 (-0.14, 0.50)	0.31 (-0.02, 0.64)	0.33 (-0.01, 0.66)	0.33 (-0.01, 0.67)
Any ε2 allele and hormonal therapy vs. no ε2 allele, control	0.12 (-0.14, 0.39)	0.11 (-0.15, 0.37)	0.14 (-0.13, 0.40)	0.15 (-0.11, 0.42)
Baseline deficits accumulation per 0.01 points	---	-0.01 (-0.02, -0.01)	-0.01 (-0.02, -0.00)	-0.01 (-0.02, -0.01)
Anxiety per 1 point	---	---	-0.00 (-0.01, 0.00)	---
Depression (depressed vs. not depressed)	---	---	---	0.03 (-0.13, 0.19)
Model Fit - BIC	2142.3	2004.8	1995.4	1964.2

[a]Results from mixed linear models; model fit was assessed using the Bayesian Information Criteria (BIC) score; lower scores indicate better fit. This primary analysis includes women with APOE ε2/2, ε2/3, or ε3/3 genotypes, grouped as having any vs no ε2 allele; women with ε2/4, ε3/4, or ε4/4 genotype are excluded (n=90 survivors and 104 controls). Each covariate adjusted for the effects of the other variables, time, interactions, and age, race, WRAT-4 score and recruitment site.

[b]The frailty item on anxiety was excluded from the baseline deficits accumulation in the model including anxiety.

[c]The frailty item on depression was excluded from the baseline deficits accumulation in the model including depression.

Supplementary Table 7.
Impact of APOE ε2 Genotype on Adjusted Longitudinal Scores on Learning and Memory Domain Controlling for Baseline Deficits Accumulation, Anxiety, Depression, or None Among Older Breast Cancer Survivors (N=330) and Non-Cancer Controls (N=317) Excluding All ε4 Carriers[a]

	Learning and Memory z-score			
	Base Model	Final Model with baseline deficits accumulation	Adding anxiety to final model[b]	Adding depression to final model[c]
	Beta (95% CI) or p-value			
APOE Genotype	p=.78	p=.70	p=.71	p=.44
Any ε2 allele vs. no ε2	0.04 (-0.17, 0.25)	0.05 (-0.16, 0.26)	0.06 (-0.15, 0.28)	0.05 (-0.16, 0.26)
Group	p=.92	p=.73	p=.77	p=.69
Chemotherapy +/- HT vs. control	0.02 (-0.16, 0.20)	-0.03 (-0.21, 0.16)	-0.01 (-0.20, 0.18)	-0.02 (-0.21, 0.17)
Hormonal vs. control	-0.01 (-0.13, 0.12)	0.02 (-0.11, 0.15)	0.02 (-0.12, 0.15)	0.04 (-0.10, 0.17)
Time	p<.001	p<.001	p<.001	p<.001
12 months vs. baseline	0.20 (0.15, 0.25)	0.20 (0.15, 0.25)	0.20 (0.15, 0.25)	0.20 (0.14, 0.25)
24 months vs. baseline	0.20 (0.15, 0.26)	0.19 (0.14, 0.25)	0.19 (0.14, 0.25)	0.19 (0.13, 0.25)
Interaction of group and genotype	p=.80	p=.92	p=.89	p=.95
Any ε2 allele and chemotherapy vs. no ε2 allele, control	-0.10 (-0.50, 0.29)	-0.07 (-0.49, 0.35)	-0.10 (-0.52, 0.33)	-0.01 (-0.44, 0.42)
Any ε2 allele and hormonal therapy vs. no ε2 allele, control	0.04 (-0.28, 0.36)	0.02 (-0.30, 0.35)	0.01 (-0.32, 0.34)	0.05 (-0.29, 0.38)
Baseline deficits accumulation per 0.01 points	---	0.00 (-0.01, 0.01)	0.00 (-0.01, 0.01)	0.00 (-0.01, 0.01)
Anxiety per 1 point	---	---	-0.01 (-0.01, 0.00)	---
Depression (depressed vs. not depressed)	---	---	---	-0.01 (-0.21, 0.20)
Model Fit - BIC	3171.6	2990.1	2970.6	2938.9

[a]Results from mixed linear models; model fit was assessed using the Bayesian Information Criteria (BIC) score; lower scores indicate better fit. This primary analysis includes women with APOE ε2/2, ε2/3, or ε3/3 genotypes, grouped as having any vs no ε2 allele; women with ε2/4, ε3/4, or ε4/4 genotype are excluded (n=90 survivors and 104 controls). Each covariate adjusted for the effects of the other variables, time, interactions, and age, race, WRAT-4 score and recruitment site.
[b]The frailty item on anxiety was excluded from the baseline deficits accumulation in the model including anxiety.
[c]The frailty item on depression was excluded from the baseline deficits accumulation in the model including depression.

Supplementary Table 8. Impact of APOE ε2 Genotype on Adjusted Longitudinal Scores on and FACT-Cog Perceived Cognitive Impairment Controlling for Baseline Deficits Accumulation, Anxiety, Depression, or None Among Older Breast Cancer Survivors (N=330) and Non-Cancer Controls (N=317) Excluding All ε4 Carriers[a]

	FACT-Cog 18-item Perceived Cognitive Impairment Score			
	Base Model	Final Model with baseline deficits accumulation	Adding anxiety to final model[2]	Adding depression to final model[3]
	Beta (95% CI) or p-value			
APOE Genotype	p=.83	p=.74	p=.58	p=.80
Any ε2 allele vs. no ε2	0.65 (-2.02, 3.32)	0.47 (-2.16, 3.09)	0.81 (-1.76, 3.38)	0.56 (-2.02, 3.14)
Group	p=.05	p=.27	p=.35	p=.51
Chemotherapy +/- HT vs. control	-1.53 (-3.83, 0.78)	-0.95 (-3.28, 1.39)	0.04 (-2.25, 2.32)	-0.15 (-2.49, 2.20)
Hormonal vs. control	-1.78 (-3.44, -0.13)	-1.01 (-2.67, 0.64)	-0.76 (-2.37, 0.86)	-0.90 (-2.54, 0.75)
Time	p=.10	p=.10	p=.08	p=.06
12 months vs. baseline	-0.62 (-1.36, 0.12)	-0.66 (-1.41, 0.08)	-0.68 (-1.43, 0.06)	-0.79 (-1.53, -0.04)
24 months vs. baseline	-0.79 (-1.57, -0.01)	-0.75 (-1.54, 0.04)	-0.82 (-1.61, -0.03)	-0.78 (-1.58, 0.01)
Interaction of group and genotype	p=.42	p=.74	p=.47	p=.67
Any ε2 allele and chemotherapy vs. no ε2 allele, control	-3.05 (-8.22, 2.11)	-2.05 (-7.29, 3.19)	-3.17 (-8.26, 1.92)	-2.34 (-7.58, 2.90)
Any ε2 allele and hormonal therapy vs. no ε2 allele, control	0.42 (-3.74, 4.58)	-0.40 (-4.50, 3.70)	-0.96 (-4.95, 3.03)	-0.14 (-4.24, 3.96)
Baseline deficits accumulation per 0.01 points	---	-0.30 (-0.39, -0.21)	-0.23 (-0.32, -0.14)	-0.22 (-0.31, -0.13)
Anxiety per 1 point	---	---	-0.32 (-0.43, -0.22)	---
Depression (depressed vs. not depressed)	---	---	---	-6.34 (-8.85, -3.83)
Model Fit - BIC	10942.5	10637.6	10480.5	10424.0

[a]Results from mixed linear models; model fit was assessed using the Bayesian Information Criteria (BIC) score; lower scores indicate better fit. This primary analysis includes women with APOE ε2/2, ε2/3, or ε3/3 genotypes, grouped as having any vs no ε2 allele; women with ε2/4, ε3/4, or ε4/4 genotype are excluded (n=90 survivors and 104 controls). Each covariate adjusted for the effects of the other variables, time, interactions, and age, race, WRAT-4 score and recruitment site.

[b]The frailty item on anxiety was excluded from the baseline deficits accumulation in the model including anxiety.

[c]The frailty item on depression was excluded from the baseline deficits accumulation in the model including depression.

Supplementary Table 9 Impact of APOE ε2 Genotype on Adjusted Longitudinal Scores on Individual Test Scores under the APE Domain Controlling for Baseline Deficits Accumulation Among Older Breast Cancer Survivors (N=412) and Non-Cancer Controls (N=407) Excluding ε2/4 Genotype[a]

	NAB Digits Forward z-score	NAB Digits Backward z-score	Trail Making A z-score	Trail Making B z-score	COWAT z-score	Digit Symbol Test z-score
	With baseline deficits accumulation					
	Beta (95% CI) or p-value					
APOE Genotype	p=.29	p=.82	p=.34	p=.38	p=.89	p=.74
Any ε2 allele vs. no ε2	-0.09 (-0.35, 0.17)	-0.15 (-0.39, 0.09)	-0.16 (-0.41, 0.09)	-0.13 (-0.38, 0.12)	-0.36 (-0.61, -0.11)	-0.11 (-0.40, 0.18)
Group	p=.71	p=.65	p=.79	p=.23	p=.95	p=1.00
Chemotherapy +/- HT vs. control	-0.07 (-0.28, 0.14)	-0.07 (-0.26, 0.12)	0.02 (-0.18, 0.23)	0.00 (-0.20, 0.21)	-0.30 (-0.50, -0.10)	-0.22 (-0.45, 0.02)
Hormonal vs. control	0.10 (-0.05, 0.24)	-0.02 (-0.15, 0.12)	-0.00 (-0.14, 0.14)	-0.05 (-0.19, 0.09)	-0.20 (-0.34, -0.06)	-0.00 (-0.17, 0.16)
Time	p=.89	p=.05	p<.001	p=.003	p<.001	p<.001
12 months vs. baseline	0.01 (-0.05, 0.08)	0.04 (-0.03, 0.10)	0.13 (0.05, 0.21)	0.12 (0.05, 0.18)	0.09 (0.04, 0.15)	0.15 (0.09, 0.21)
24 months vs. baseline	0.01 (-0.05, 0.08)	0.09 (0.02, 0.16)	0.16 (0.08, 0.25)	0.08 (0.01, 0.15)	0.17 (0.12, 0.23)	0.20 (0.14, 0.27)
Interaction of group and genotype	p=.90	p=.35	p=.81	p=.21	p=.02	p=.33
Any ε2 allele and chemotherapy vs. no ε2 allele, control	0.02 (-0.49, 0.54)	0.33 (-0.15, 0.81)	0.07 (-0.44, 0.58)	0.43 (-0.07, 0.93)	0.66 (0.16, 1.15)	0.43 (-0.16, 1.01)
Any ε2 allele and hormonal therapy vs. no ε2 allele, control	-0.08 (-0.49, 0.32)	0.18 (-0.20, 0.55)	0.13 (-0.26, 0.52)	0.22 (-0.18, 0.61)	0.38 (-0.01, 0.76)	0.01 (-0.44, 0.47)
Baseline deficits accumulation per 0.01 points	-0.00 (-0.01, 0.01)	0.00 (-0.00, 0.01)	-0.02 (-0.03, -0.01)	-0.01 (-0.02, -0.01)	-0.01 (-0.02, 0.00)	-0.02 (-0.03, -0.02)
Model Fit - BIC	4829.7	4852.3	5260.2	4928.5	4341.1	4878.4

[a]Results from mixed linear models; model fit was assessed using the Bayesian Information Criteria (BIC) score; lower scores indicate better fit. This primary analysis includes women with APOE ε2/2, ε2/3, ε3/3, ε3/4, or ε4/4 genotypes, grouped as having any vs. no ε2 allele; women with ε2/4 genotype are excluded (n=8 survivors and 14 controls). Each covariate adjusted for the effects of the other variables, time, interactions, and age, race, WRAT-4 score and recruitment site.

Supplement Table 10 Impact of APOE ε2 Genotype on Adjusted Longitudinal Scores on Objective Cognition Test Domains Controlling for Baseline Cardiovascular Comorbidities Among Older Breast Cancer Survivors (N=412) and Non-Cancer Controls (N=407) Excluding ε2/4 Genotype[a]

	Attention, Processing Speed and Executive Functioning z-score	Learning and Memory z-score	FACT-Cog 18-item Perceived Cognitive Impairment Score
	Base Model with baseline cardiovascular comorbidities including hypertension		
	Beta (95% CI) or p-value		
APOE Genotype	p=.64	p=.58	p=.84
Any ε2 allele vs. no εe2	-0.15 (-0.32, 0.01)	0.05 (-0.16, 0.25)	0.89 (-1.70, 3.47)
Group	p=.97	p=.74	p=.03
Chemotherapy +/- HT vs. control	-0.11 (-0.24, 0.02)	-0.05 (-0.21, 0.12)	-1.64 (-3.71, 0.42)
Hormonal vs. control	-0.05 (-0.14, 0.04)	0.00 (-0.11, 0.12)	-1.55 (-3.01, -0.10)
Time	p<.001	p<.001	p=.07
12 months vs. baseline	0.09 (0.06, 0.12)	0.20 (0.15, 0.24)	-0.61 (-1.27, 0.05)
24 months vs. baseline	0.13 (0.09, 0.16)	0.19 (0.14, 0.24)	-0.76 (-1.46, -0.05)
Interaction of group and genotype	p=.30	p=.95	p=.44
Any ε2 allele and chemotherapy vs. no ε2 allele, control	0.24 (-0.09, 0.56)	-0.04 (-0.44, 0.36)	-3.19 (-8.28, 1.90)
Any ε2 allele and hormonal therapy vs. no ε2 allele, control	0.13 (-0.12, 0.39)	0.03 (-0.29, 0.35)	-0.08 (-4.15, 3.99)
Baseline cardiovascular comorbidities including hypertension per 1 comorbidity condition	-0.02 (-0.08, 0.04)	0.05 (-0.02, 0.12)	-0.21 (-1.12, 0.71)
Model Fit - BIC	2536.8	3760.0	13489.3

[a]Results from mixed linear models; model fit was assessed using the Bayesian Information Criteria (BIC) score; lower scores indicate better fit. This primary analysis includes women with APOE ε2/2, ε2/3, ε3/3, ε3/4, or ε4/4 genotypes, grouped as having any vs no ε2 allele; women with ε2/4 genotype are excluded (n=8 survivors and 14 controls). Each covariate adjusted for the effects of the other variables, time, interactions, and age, race, WRAT score and recruitment site. The inclusion of cardiovascular disease (instead of deficits accumulation scores) did not improve model fit so were not used in the final models in Table 3.

Supplementary table 11 adjusted mean attention, processing speed, and executive functioning z-scores over time

	Sample Size (N=766)	Adjusted mean attention, processing speed, and executive functioning z-score (95% CI), [Sample size]		
		Baseline	12 Months	24 Months
Figure 2: Survivors and Controls by E2 Positivity and Time				
Chemotherapy +/- hormonal, any ε2 (excluding ε2/ε4 genotype)	18	-0.05 (-0.31, 0.20), [18]	0.04 (-0.22, 0.29), [13]	0.07 (-0.19, 0.33), [11]
Chemotherapy +/- hormonal, no ε2 allele	85	-0.21 (-0.34, -0.09), [85]	-0.12 (-0.25, 0.00), [67]	-0.09 (-0.22, 0.04), [56]
Hormonal, any ε2 (excluding ε2/ε4 genotype)	35	-0.17 (-0.36, 0.01), [35]	-0.08 (-0.27, 0.10), [29]	-0.05 (-0.23, 0.13), [22]
Hormonal, no ε2 allele	240	-0.14 (-0.22, -0.06), [240]	-0.05 (-0.13, 0.04), [188]	-0.02 (-0.10, 0.07), [159]
Control, any ε2 (excluding ε2/ε4 genotype)	48	-0.27 (-0.43, -0.11), [48]	-0.18 (-0.34, -0.02), [46]	-0.15 (-0.31, 0.01), [37]
Control, no ε2 allele	340	-0.11 (-0.18, -0.04), [340]	-0.02 (-0.09, 0.06), [305]	0.01 (-0.06, 0.09), [255]

Supplementary table 12 adjusted mean learning and memory z-scores over time

	Sample Size (N=766)	Adjusted mean learning and memory z-score (95% CI), [Sample size]		
		Baseline	12 Months	24 Months
Figure 3: Survivors and Controls by E2 Positivity and Time				
Chemotherapy +/- hormonal, any e2 (excluding ε2/ε4 genotype)	18	-0.18 (-0.51, 0.15), [18]	0.02 (-0.31, 0.35), [13]	0.01 (-0.32, 0.34), [11]
Chemotherapy +/- hormonal, no ε2 allele	85	-0.18 (-0.34, -0.02), [85]	0.02 (-0.14, 0.18), [67]	0.01 (-0.15, 0.17), [56]
Hormonal, any ε2 (excluding ε2/ε4 genotype)	35	-0.05 (-0.28, 0.18), [35]	0.14 (-0.09, 0.38), [29]	0.14 (-0.10, 0.37), [22]
Hormonal, no ε2 allele	240	-0.12 (-0.23, -0.01), [240]	0.08 (-0.03, 0.19), [188]	0.07 (-0.04, 0.18), [159]
Control, any ε2 (excluding ε2/ε4 genotype)	48	-0.08 (-0.28, 0.12), [48]	0.12 (-0.08, 0.32), [46]	0.11 (-0.09, 0.31), [37]
Control, no ε2 allele	340	-0.13 (-0.22, -0.03), [340]	0.07 (-0.02, 0.17), [305]	0.06 (-0.03, 0.16), [255]

Supplementary table 13 post-hoc group comparisons of learning and memory z-scores

Post-hoc group comparisons		
ε2+ chemo vs. ε2- chemo	---	0.00 (-0.35, 0.35), p=1.00
ε2+ hormonal vs. ε2- hormonal	---	0.07 (-0.18, 0.31), p=.59
ε2+ control vs. ε2- control	---	0.05 (-0.16, 0.25), p=.65
ε2+ chemo vs. ε2+ hormonal	---	-0.13 (-0.52, 0.27), p=.53
ε2+ chemo vs. ε2- hormonal	---	-0.06 (-0.39, 0.28), p=.73
ε2+ chemo vs. ε2+ control	---	-0.10 (-0.48, 0.28), p=.60
ε2+ chemo vs. ε2- control	---	-0.05 (-0.38, 0.28), p=0.76
